# Implementation of a combined CDK inhibition and arginine-deprivation approach to target arginine-auxotrophic glioblastoma multiforme cells

**DOI:** 10.1038/s41419-022-05006-1

**Published:** 2022-06-18

**Authors:** Christin Riess, Katharina del Moral, Adina Fiebig, Philipp Kaps, Charlotte Linke, Burkhard Hinz, Anne Rupprecht, Marcus Frank, Tomas Fiedler, Dirk Koczan, Sascha Troschke-Meurer, Holger N. Lode, Nadja Engel, Thomas Freitag, Carl Friedrich Classen, Claudia Maletzki

**Affiliations:** 1 University Children’s Hospital, Rostock University Medical Center, Ernst-Heydemann-Straße 8, 18057 Rostock, Germany; 2 Department of Medicine, Clinic III - Hematology, Oncology, Palliative Medicine, Rostock University Medical Center, Ernst-Heydemann-Straße 6, 18057 Rostock, Germany; 3 Institute for Medical Microbiology, Virology, and Hygiene, Rostock University Medical Center, Schillingallee 70, 18057 Rostock, Germany; 4 Institute for Pharmacology and Toxicology, Rostock University Medical Center, Schillingallee 70, 18057 Rostock, Germany; 5 Medical Biology and Electron Microscopy Center, Rostock University Medical Center, Rostock, Germany; 6 Department of Life, Light & Matter, University of Rostock, Rostock, Germany; 7 Institute for Immunology, University of Rostock, 18055 Rostock, Germany; 8 Department of Pediatric Oncology and Hematology, University Medicine Greifswald, Ferdinand-Sauerbruch-Strasse, 17475 Greifswald, Germany; 9 Department of Oral and Maxillofacial Surgery, Facial Plastic Surgery, Rostock University Medical Center, Rostock, Germany

**Keywords:** CNS cancer, DNA damage and repair, Cell death

## Abstract

Constitutive activation of cyclin-dependent kinases (CDKs) or arginine auxotrophy are hallmarks of Glioblastoma multiforme (GBM). The latter metabolic defect renders tumor cells vulnerable to arginine-depleting substances, such as arginine deiminase from *Streptococcus pyogenes* (SpyADI). Previously, we confirmed the susceptibility of patient-derived GBM cells towards SpyADI as well as CDK inhibitors (CDKis). To improve therapeutic effects, we here applied a combined approach based on SpyADI and CDKis (dinaciclib, abemaciclib). Three arginine-auxotrophic patient-derived GBM lines with different molecular characteristics were cultured in 2D and 3D and effects of this combined SpyADI/CDKi approach were analyzed in-depth. All CDKi/SpyADI combinations yielded synergistic antitumoral effects, especially when given sequentially (SEQ), i.e., CDKi in first-line and most pronounced in the 3D models. SEQ application demonstrated impaired cell proliferation, invasiveness, and viability. Mitochondrial impairment was demonstrated by increasing mitochondrial membrane potential and decreasing oxygen consumption rate and extracellular acidification rate after SpyADI/abemaciclib monotherapy or its combination regimens. The combined treatment even induced autophagy in target cells (abemaciclib/SpyADI > dinaciclib/SpyADI). By contrast, the unfolded protein response and p53/p21 induced senescence played a minor role. Transmission electron microscopy confirmed damaged mitochondria and endoplasmic reticulum together with increased vacuolization under CDKi mono- and combination therapy. SEQ-abemaciclib/SpyADI treatment suppressed the DSB repair system via NHEJ and HR, whereas SEQ-dinaciclib/SpyADI treatment increased γ-H2AX accumulation and induced Rad51/Ku80. The latter combination also activated the stress sensor GADD45 and β-catenin antagonist AXIN2 and induced expression changes of genes involved in cellular/cytoskeletal integrity. This study highlights the strong antitumoral potential of a combined arginine deprivation and CDK inhibition approach via complex effects on mitochondrial dysfunction, invasiveness as well as DNA-damage response. This provides a good starting point for further in vitro and in vivo proof-of-concept studies to move forward with this strategy.

## Abstract (web summary)

The complex effects of combined CDki/SPyADI application on arginine-auxotrophic glioblastoma multiforme cells. CDki/SPyADI combination therapy impairs cell proliferation, invasiveness, gene expression, induces mitochondrial impairment, and vacuole formation. Abemaciclib-SPyADI-treatment suppresses DNA repair, dinaciclib-SpyADI-treatment enhances γ-H2AX accumulation and activates the stress sensor GADD45 and β-catenin antagonist AXIN2. Both CDKi/SpyADI combinations significantly boost cell death.

## Graphical abstract

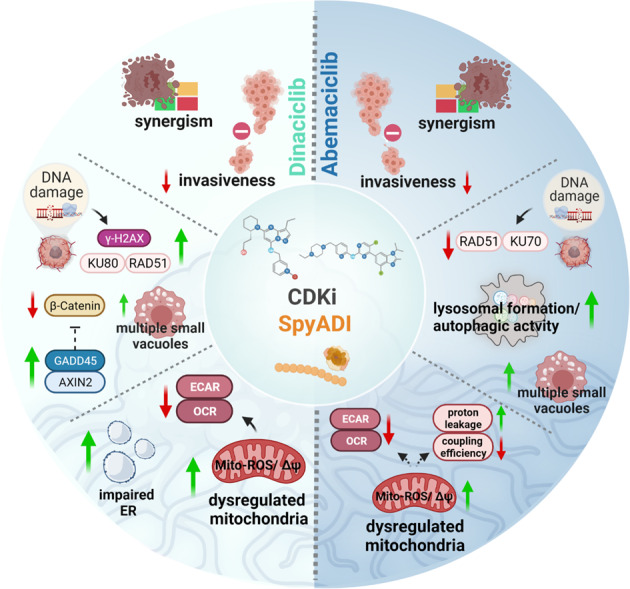

## Introduction

Metabolic dysregulation is a hallmark of cancer, characterized by changes in metabolic pathways for energy production and biosynthetic processes [1]. To sustain growth, tumor cells have a higher demand for nutrients than normal cells and in some cases, they become auxotrophic to certain amino acids. This inability to produce specific nutrients themselves contributes to the dependence on exogenous supply from adjacent normal cells. A quite prominent example is arginine, one of the conditionally essential amino acids playing a pivotal role in cellular division and metabolism [2]. Mutations and epigenetic modifications in the genes responsible for arginine biosynthesis (argininosuccinate synthetase 1 and argininosuccinate lyase) [3–5] contribute to arginine auxotrophy. Arginine deiminase (ADI) is a bacterial enzyme capable of degrading l-arginine into citrulline and ammonia. This finding attracted much attention in cancer therapy with positive outcomes from (pre-)clinical trials upon specific depletion of systemic arginine by pegylated ADI during the last years [6–9]. In 2019, a phase I study on ASS1-deficient recurrent and highly pretreated high-grade glioblastoma multiforme (GBM) patients confirmed safety and induction of stable disease in 80% of patients receiving a PEG-ADI [10]. Our preclinical studies on *Streptococcus pyogenes* arginine deiminase (SpyADI) additionally revealed growth inhibition by induction of cellular stress-responses, autophagocytosis, and senescence in GBM cells [11]. Hence, arginine-deprivation strategies should be considered further for implementation into clinical practice. To prevent resistance, SpyADI may be combined with targeted agents. A quite promising class of targeted agents belongs to the cyclin-dependent kinase inhibitors (CDKis). Most cancers exhibit molecular-driven constitutive activation of CDKs. These regulatory checkpoints drive all cell cycle transitions. Indeed, inhibitors blocking specific cell cycle regulation were already approved by the FDA for the treatment of advanced-stage or metastatic, hormone-receptor-positive, HER2-negative breast cancer [12–14]. The hallmark of genomic instability in GBM is related to perturbations in the S-phase and G2/M transition, with the cyclin D1-CDK4/6-Rb pathway being mostly altered [15]. Just recently, we described the successful elimination of patient-derived two-dimensional (2D)- and three-dimensional (3D)-cultured GBM cells by the global CDKi dinaciclib as well as the selective CDK4/6 inhibitor abemaciclib [16, 17]. The aforementioned CDKi even interfered with the tryptophan catabolism and had complex effects at both the morphological and molecular levels [17].

Given the high clinical relevance of CDK pathway alterations in GBM and the high level of arginine-auxotrophy in these tumors, we here combined CDKis with arginine-deprivation therapy to boost overall response. We identified sequential schedules to be most effective in GBM treatment. Cytotoxic effects included induction of autophagy, mitochondrial impairment, and dysregulated DNA repair that shed light on the molecular effects driven by this combination.

## Results

### Dinaciclib synergizes with SpyADI to inhibit GBM cell growth

**Fig. 1:**
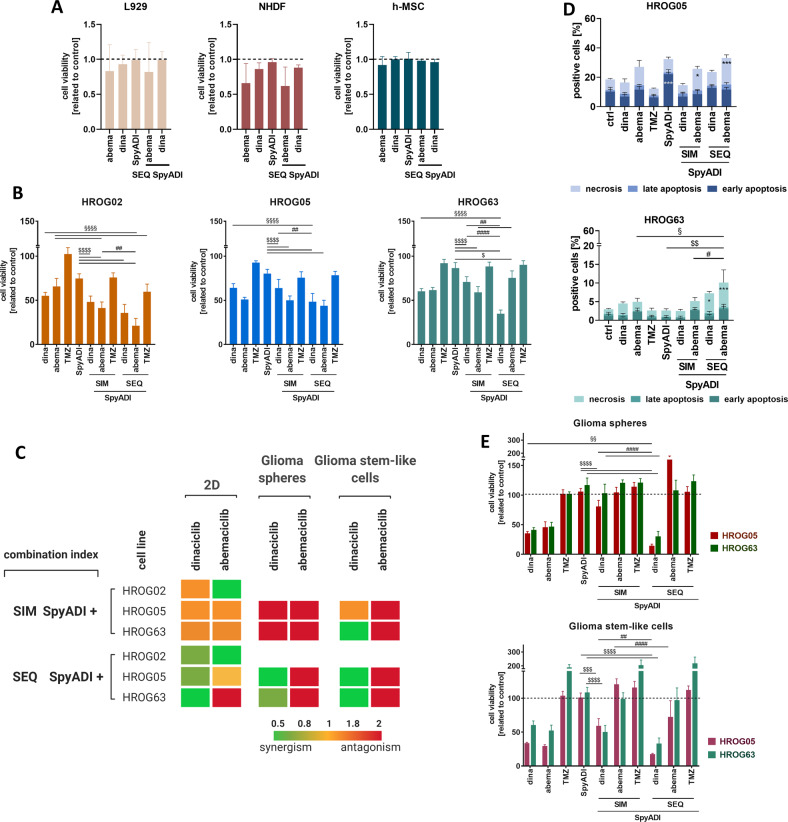
Viability and cytotoxicity assays after mono- and combination therapy using different settings and experimental conditions. **A** Viability of non-malignant control cells. Cells (L929, NHDF, and h-MSCs) were treated with the respective substances for 2 × 72 h in a sequential treatment regimen (SEQ). Quantitative analysis was done using Calcein AM assay. *n* = 3 independent experiments. **B**–**E** GBM cells were treated with the respective substances for 2 × 72 h in a simultaneous or sequential treatment regimen (SIM; SEQ). **B** Quantitative analysis of cell viability using Calcein AM in the 2D-model (HROG02, HROG05, HROG63). *n* = 8 independent experiments. **C** Bliss independence calculation. The Bliss independence model was applied to determine synergistic, additive or antagonistic effects based on the results obtained after 2 x 72 h treatment with the indicated substances. Interpretation is as follows: Δ < 1: synergism (green pattern); Δ = 1: additive (yellow pattern); Δ > 1: antagonistic (orange and red pattern). Image was created with Biorender.com. **D** Quantitative analysis of cytotoxicity was done via flow cytometry upon staining with Yo-Pro1 and PI. Cells were either characterized as early-apoptotic, late-apoptotic, or necrotic. *n* = 3 independent experiments; statistical differences were obtained for the necrotic cell fraction: *p < 0.05; ***p < 0.001; §*p* < 0.05; $$*p* < 0.01; #*p* < 0.05. **E** Quantitative analysis of cell viability in the 3D systems (HROG05, HROG63) was done using 3D-Glo. **B**, **E** Viability reduction (%) after treatment was quantified by normalization to control values (untreated cells set to be 100 %). *n* = 8 independent experiments; §§*p* < 0.01; §§§§*p* < 0.0001; $*p* < 0.05; $$$*p* < 0.001; $$$$*p* < 0.0001; ##*p* < 0.01; ####*p* < 0.0001. One-way ANOVA.

Two-dimensional and 3D-cultured patient-derived GBM cell lines HROG02, HROG05, and HROG63 [11, 18] were treated simultaneously (SIM) and sequentially (SEQ) for two rounds (144 h) (Fig. 1). Non-malignant control cells (fibroblasts, mesenchymal stem cells) were included in viability assays to assess the impact of the therapy regimens on normal cells (Fig. 1A). Notably, these control cells were only marginally affected by either treatment, providing a ready basis for preclinical testing.

CDKi monotherapy remarkably reduced the GBM cell number and induced cell shrinkage in 2D-culture compared to Temozolomide (TMZ) and SpyADI monotherapy (Fig. 1B and SFig. 1A). In simultaneous combinations, cytostatic effects were only seen after combined abemaciclib/SpyADI therapy (HROG02, Fig. 1B, C). This regimen induced morphological changes, characterized by partial enlargement, accompanied by a flattened structure. Using the sequential approach, cells were treated with TMZ or CDKis in first-line, followed by SpyADI. Here, SEQ-dinaciclib/SpyADI was always synergistic. SEQ-abemaciclib/SpyADI revealed synergistic and additive effects in HROG02/05, but not in HROG63. In HROG02/05, cell shrinkage was seen and resulted in significantly reduced cell viability (Fig. 1B and SFig. 1A). TMZ/SpyADI reached only antagonistic effects (Fig. 1C). In complementary cytotoxicity assays, apoptosis and necrosis was determined (Fig. 1D). This analysis confirmed the therapeutic activity of CDKi/SpyADI combinations—especially in the SEQ-setting (*p* < 0.001 vs. control and *p* < 0.01 vs. monotherapy and *p* < 0.05 vs. SIM-CDKi/SpyADI). In here, SEQ-abemaciclib/SpyADI induced massive necrosis in GBM cells, which even exceeded the effects of SEQ-dinaciclib/SpyADI treatment. TMZ had no impact on viability, which is in line with the HROGs MGMT promoter methylation status (HROG05: < 35 %; HROG63: unmethylated), contributing to TMZ resistance.

In the 3D-spheroids, HROG05/63 were grown as glioma spheres and glioma stem-like cells. SIM-CDKi/SpyADI revealed antagonistic effects in glioma spheres (Fig. 1C, E and SFig. 1B). SIM-dinaciclib/SpyADI was synergistic in glioma stem-like cells of HROG63. SEQ-dinaciclib/SpyADI, but not SEQ-abemaciclib/SypADI synergistically potentiated anti-tumor effects of the monotherapies in both spheroid models (Fig. 1C, E). Notably, effects were much stronger than in the 2D-model. TMZ + /- SpyADI did not affect 3D-cultures.

To sum up, the sequential treatment is superior to the simultaneous approach, notably, both in 2D and 3D models. Consequently, we focused on the sequential setting and on CDKis as SpyADI combination partners to treat HROG05 and HROG63 in further experiments.

### CDKi/SpyADI induce gross morphological changes

**Fig. 2:**
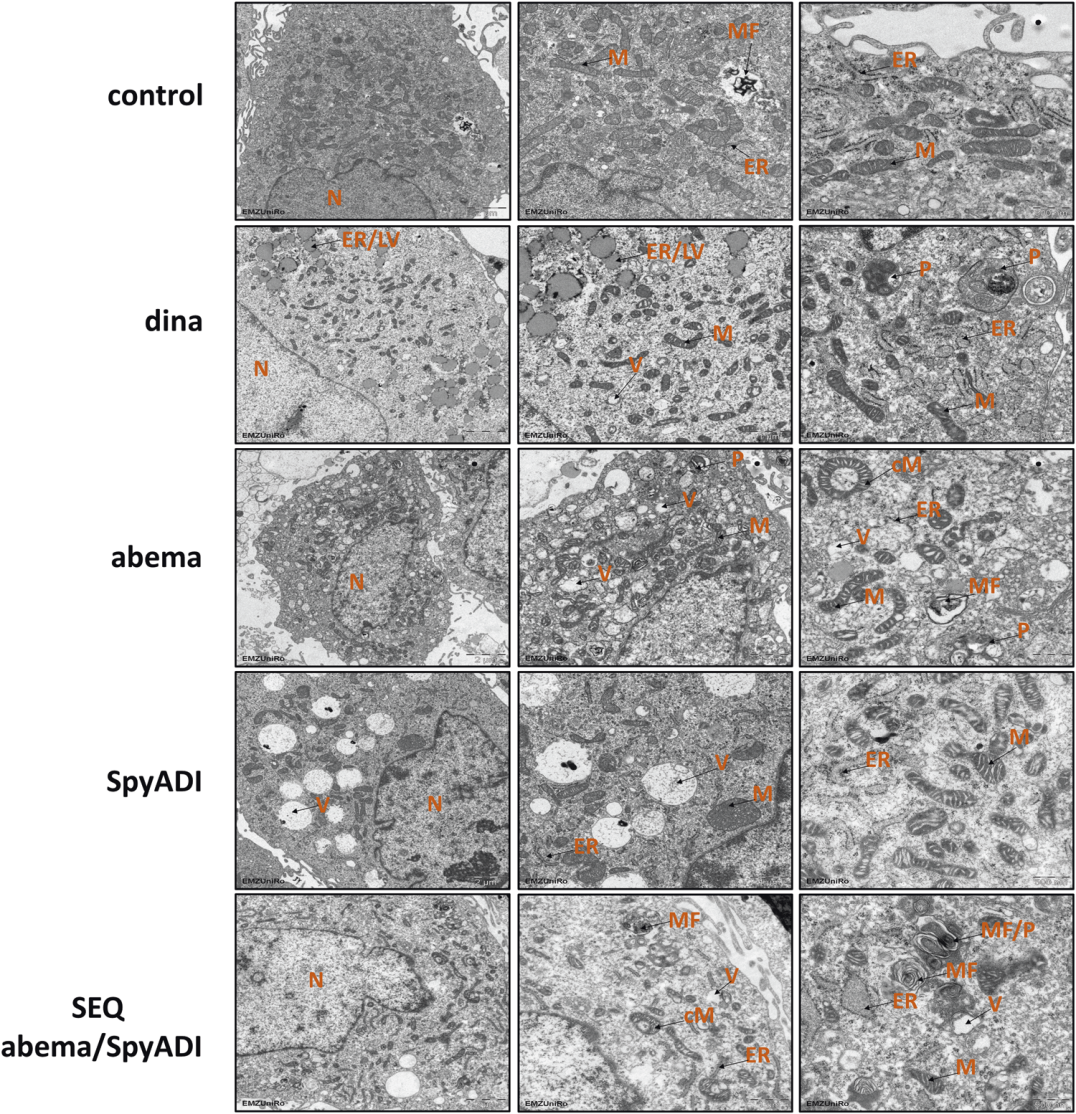
Transmission electron microscopy. Pellets of HROG05 cells were harvested after the indicated treatments and embedded for electron microscopy as described in Materials and Methods. Ultrastructural changes were examined, focusing on ER and mitochondrial morphology, signs of autophagy, and processes associated with cell death. Shown are representative images of untreated cells compared to various treated cells (scale bars: 1–2 µm). The left image panel provides an overview of the treated cells in each case (scale bar: 2 µm) with higher magnification shown in the middle panel (scale bar 1 µm), respectively. Images on the right provide additional information about treatment-specific effects, such as vacuoles, altered structure of mitochondria or abnormal ER (scale bar: 500 nm). ER endoplasmic reticulum, M mitochondria, cM circular mitochondria, MF myelin figure, LV lipid vesicle, N nucleus, P phagosome, V vacuole. Sections were examined using a Zeiss EM902 electron microscope at 80 kV. Digital images were acquired with a side-mounted 1x2k FT-CCD camera.

To gain insights into CDKi/SpyADI induced morphological changes and cellular dynamics in HROG05, we performed transmission electron microscopy (TEM) analyses. TEM confirmed multiple ultrastructural changes, including structural damage and autophagy of mitochondria as well as altered endoplasmic reticulum (ER) morphology (Fig. 2). In addition, vacuolization remarkably increased in SpyADI treated cells and, to a lesser extent, upon dinaciclib and abemaciclib. Some of these vacuoles were filled with debris resembling remains of organelles. Alteration and disintegration of the ER were most pronounced with dinaciclib and small vesicles with lipid droplet-like appearance were commonly observed with this treatment. Mitochondrial damage included swollen mitochondria and disintegrated cristae (e.g., SpyADI and dinaciclib). More severe damage involving autophagy was evident in both abemaciclib-mono- and in combination therapy with SpyADI (Fig. 2). Thus, numerous phagosomes were seen at some sites with myelin-like inclusions, representing complex, nested vesicles derived from either the lysosomal degradation of mitochondria and/or other organelles.

### CDKi/SpyADI therapy impairs colony formation ability and invasiveness

**Fig. 3:**
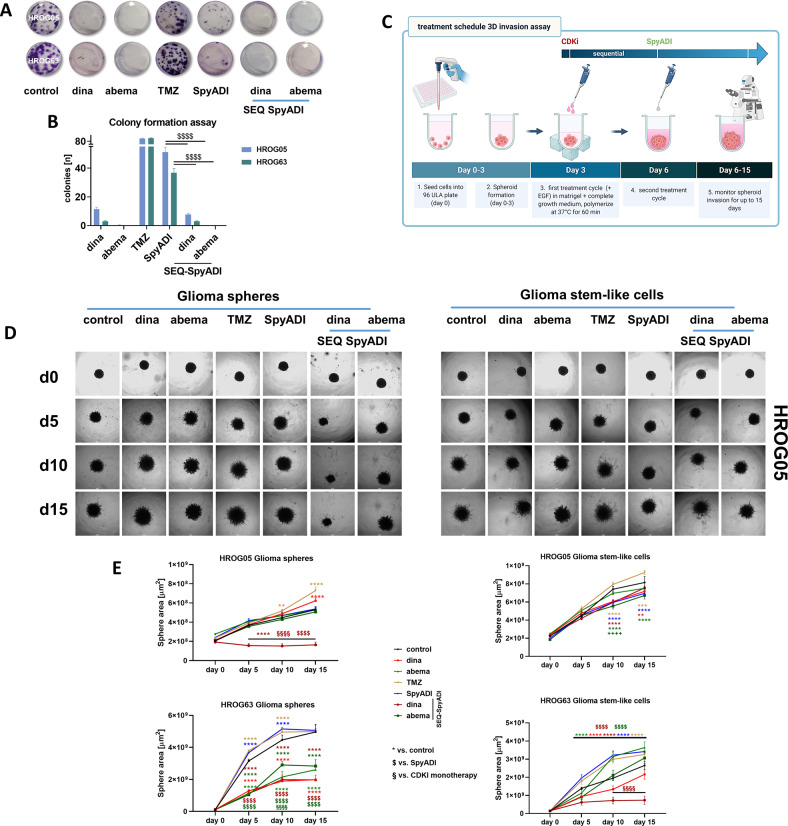
Colony formation and tumor-spheroid invasion assay. **A**, **B** The clonogenic potential of 2D-cultured GBM cells was examined by seeding cells at a density of 1.0 × 10^3^ per well. Thereafter, cells were treated for a total of 2 × 72 h and cultured for additional 14 days without treatment (=recovery). Biomass was quantified upon staining with 0.2% crystal violet and images were taken with a Leica DMI 4000B. **A** Representative light microscopic images. **B** Quantification of biomass (=cell colonies) using ImageJ software (colony count of treated cells; control >100 colonies/well, not presented). *n* = 3 independent experiments; *p < 0.0001; $*p* < 0.05; $$*p* < 0.01. One-way ANOVA. **C** Treatment schedule for the 3D invasion assay. **D**, **E** Analysis of invasive capacity into a matrigel-matrix. 3D-cultured GBM cells were treated with indicated substances or left untreated. GBM cells were monitored for a total of 15 days. Images were taken at a 5-day interval using a Leica DMI 4000B microscope (scale bar **A**, **B**: 50 μm). **D** Representative images of HROG05 Glioma spheres (left side) and Glioma stem-like cells (right side). **E** Invasive capacity (area of the spheres [µm^2^]) was examined with the oval selection function of FIJI-ImageJ according to [64]. *p < 0.05, **p < 0.01, ****p < 0.0001; $$$$*p* < 0.0001; §§§§*p* < 0.0001 dinaciclib; $$$$*p* < 0.0001. Two-way ANOVA.

The ability of our GBM cell lines to form colonies during treatment (2 × 72 h) and after recovery (14 day) was analyzed. Colony formation was massively impaired by CDKi or SpyADI and completely inhibited by SEQ-CDKi/SpyADI (Fig. 3A, B). We then implanted defined glioma spheres and glioma stem-like cells (*n* = 1 spheroid/well, triplicates) of both cell lines into matrigel and monitored sphere outgrowth under CDKi/SpyADI therapy (Fig. 3C). Untreated and TMZ-treated cells showed the expected high basal invasiveness. Sphere outgrowth under CDKi/SpyADI treatment was impaired, especially under SEQ-dinaciclib/SpyADI therapy (Fig. 3D, E). The HROG05 glioma spheres and notably HROG63 glioma stem-like cells even began to shrink, confirming the substantial toxicity of the regimen. To further analyze this and to determine whether the reduced invasiveness was due solely to toxicity or also to impaired invasiveness, we performed the matrigel-invasion assay with HROG63 glioma spheres stably transduced with a lentiviral vector expressing the near-infrared fluorescent protein iRFP680 (NIR-680) (sFig. 2). This method allows simultaneous quantification of viability and invasiveness (sFig. 2B). Using HROG63 glioma spheres, we identified impaired invasiveness at early time points (<day 10) under CDKI mono- and SEQ-CDKI/SpyADI combination therapy. This finally contributed to cell death, as detected by reduced fluorescence intensity later on (day 15, sFig. 2C). Hence, we conclude a combined anti-invasive and toxic effect on tumor cells, which is most effective when a SEQ-CDKI/SpyADI treatment is used (sFig. 2).

### CDKi/SpyADI therapy trigger cellular stress responses

**Fig. 4:**
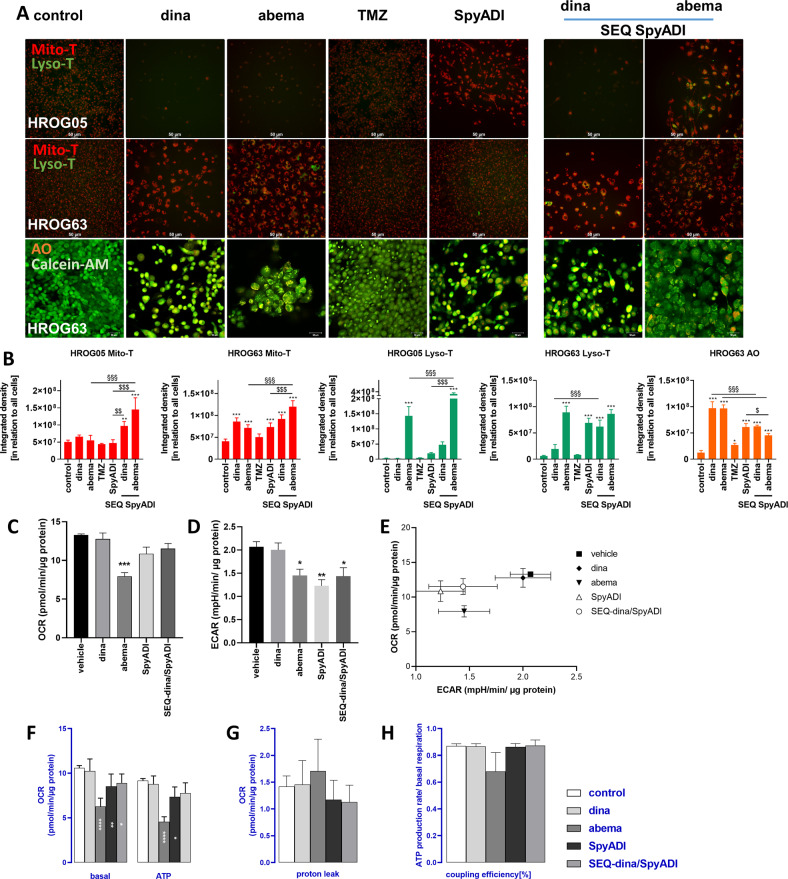
Influence on mitochondria, lysosomes/autophagy, ER and extracellular flux analysis. **A**, **B** Mitochondrial function and/or acidic compartments (LysoTracker and Acridine orange (AO)) in 2D-cultured and treated GBM cells (HROG05, HROG63) was examined by immunofluorescence imaging as described in materials and methods (Mito- [red] and Lyso-Tracker [green]; Calcein AM [green] and Acridine orange [orange]). **A** Merged fluorescence is presented (scale bar **A**: 50 μm). CDKi-SpyADI impairs mitochondrial membrane potential (MMP) and SEQ-abemaciclib administration elevates LysoTracker and AO staining. **B** Quantification of Mito- and Lyso-Tracker as well as AO-positive cells using ImageJ software (integrated density analysis). *n* = 3–6 independent experiments; **p < 0.01; ***p < 0.001; §§§*p* < 0.001; $*p* < 0.05; $$*p* < 0.01; $$$*p* < 0.001. One-way ANOVA. **C**–**H** The oxygen consumption rate (OCR) and extracellular acidification rate (ECAR) of treated HROG05 cells was recorded by a Seahorse XFe24 Analyzer (**C**, **D**). To determine the basal respiration (basal, **E**), ATP-dependent respiration (ATP, **F**), proton leak (**G**) and coupling efficiency (**H**) mitochondrial stress test was applied with injection of 1.5 µM oligomycin (Port A), 1.0 µM FCCP (Port B) and 0.5 µM Antimycin A and Rotenon, each (Port C). Decreased OCR and ECAR after treatment with abemaciclib, SpyADI or SEQ dinaciclib/SpyADI were observed. Significant reduction of basal OCR and partially also of ATP-production-based OCR was shown, indicating dysfunctional mitochondria. *n* = 3 independent experiments; ∗*p* < 0.05; ∗∗*p* < 0.002; ∗∗∗*p* < 0.0002; ∗∗∗∗*p* < 0.0001. One-way ANOVA.

Interference of CDKi/SpyADI with mitochondria (increased mitochondrial reactive oxygen species (ROS) production and mitochondrial membrane potential) and lysosomal formation were studied based on our previous observations under CDKi monotherapy [16]. Mitochondrial activity increased cell line-specific after SEQ-dinaciclib/SpyADI or SEQ-abemaciclib/SpyADI treatment (Fig. 4A, B). Likewise, lysosome formation and autophagic activity were observed, notably in SEQ-abemaciclib/SpyADI (Fig. 4A, B and SFig. 3). Abemaciclib and its combination and TMZ tended to increase the abundance of the autophagy-related proteins LC3A/B, Beclin-1, ATG3, and ATG5n (SFig. 3). Since no such alterations were seen in SEQ-dinaciclib/SpyADI-treated cells, we conclude a selective SEQ-abemaciclib/SpyADI-induced autophagic response.

The finding of abnormal mitochondria in the TEM images and increased mitochondrial membrane potential, prompted us to further investigate the real-time oxygen consumption rate (OCR) in GBM cells using a Seahorse Extracellular Flux (XF24) analyzer. OCR (Fig. 4C) and ECAR (Fig. 4D) decreased after treatment with abemaciclib, SpyADI or SEQ-dinaciclib/SpyADI. This is also reflected in the energy metabolism profile by the shift to a less energetic state (Fig. 4E). The application of a MitoStressTest allowed the calculation of basal respiration and ATP-production-dependent respiration (Fig. 4F). Significant reduction of basal OCR and partially also of ATP-production-based OCR was shown, indicating dysfunctional mitochondria. The abemaciclib-treated cells also showed increased mitochondrial proton leakage (Fig. 4G), which resulted in reduced coupling efficiency (Fig. 4H). Both are clear signs of mitochondrial damage.

**Fig. 5:**
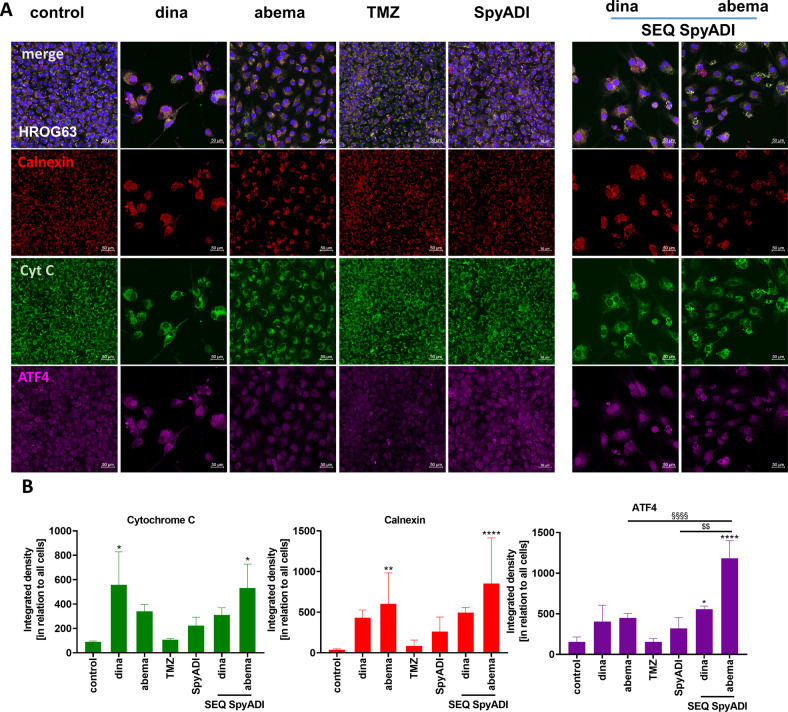
Detection and quantification of cytochrome c, calnexin, and ATF-4. **A** GBM cells (HROG63) were stained with respective antibodies to determine the subcellular localization of cytochrome c (green), calnexin (red), ATF-4 (violet) and counterstained with DAPI to visualize nuclei (blue). CDKi-SpyADI combination resulted in minor UPR. Merged images as well as single images were taken on a Leica DMI 4000B for mitochondrial function/acidic compartment evaluation and ER-stress marker and cytochrome c localization were analyzed via a Zeiss Elyra 7 Confocal Laser Microscope. Representative images are shown. **B** Quantification of specific markers. *n* = 3 independent experiments; *p < 0.05, **p < 0.01; ****p < 0.0001; §§§§*p* < 0.001; $$*p* < 0.01. One-way ANOVA.

Alteration and disintegration of the ER was observed during the TEM course. To further assess whether ER stress is associated with the CDKi-SpyADI-induced cytotoxicity in GBM cells, we examined abundance of calnexin, ATF4, and cytochrome c (Fig. 5). In CDKi-mono- and SEQ-CDKi/SpyADI-treated cells, abundance of calnexin and ATF4 as well as redistribution of cytochrome c increased, which reached statistical significance in SEQ-abema/SpyADI treatment. Notably, levels of ATF4 were even higher than in the respective monotherapy approach, i.e., abema or SpyADI (Fig. 5B). Hence, involvement of a ER stress response under treatment is very likely.

### Induction of senescence and DNA damage by mono- and combinational CDKi/SpyADI treatment

DNA damage, such as DNA double-strand breaks, triggers senescence. Senescent cells are characterized by a persistent DNA-damage response (DDR) through activation of p53/p21 and p16 pathways as well as β-galactosidase activity induced by chemotherapeutic, oxidative, or genotoxic stress. Hence, we examined the activity of β-galactosidase, as well as activation of p16/p21/p53 and formation γ-H2AX foci as markers of senescence and DNA double-strand breaks in GBM cells. Additionally, the impact on the Wnt/β-catenin-signaling-pathway was studied.

The induction of β-galactosidase was cell line-specific. Generally, CDKi/SpyADI combinations triggered senescence more effectively than monotherapy (SFig. 4). DDR proteins p21 and p53 were occasionally detectable in the nucleus of controls. Amounts increased in SEQ-dinaciclib/SpyADI, while SEQ-abemaciclib/SpyADI tended to reduce the abundance of these proteins. The third senescence-associated marker p16 was detected in the nucleus and cytoplasm of the cells. Its abundance remained nearly unaffected under treatment. The only exception was SEQ-abemaciclib/SpyADI, in which p16 was downregulated (SFig. 4).

**Fig. 6:**
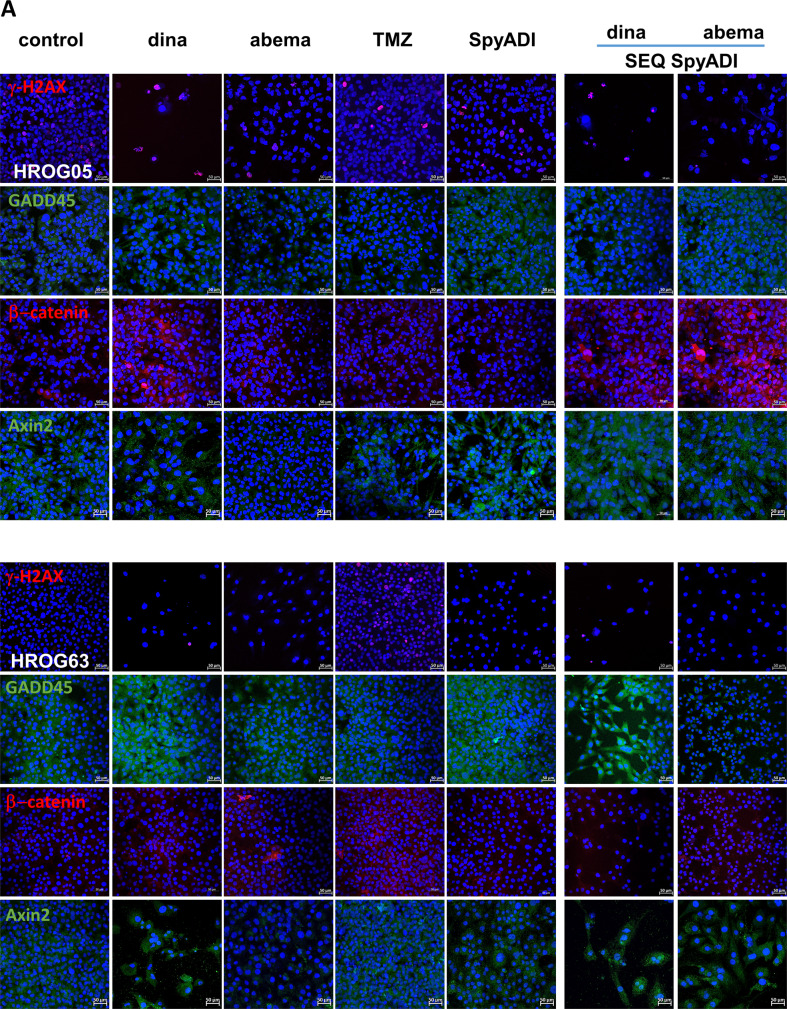

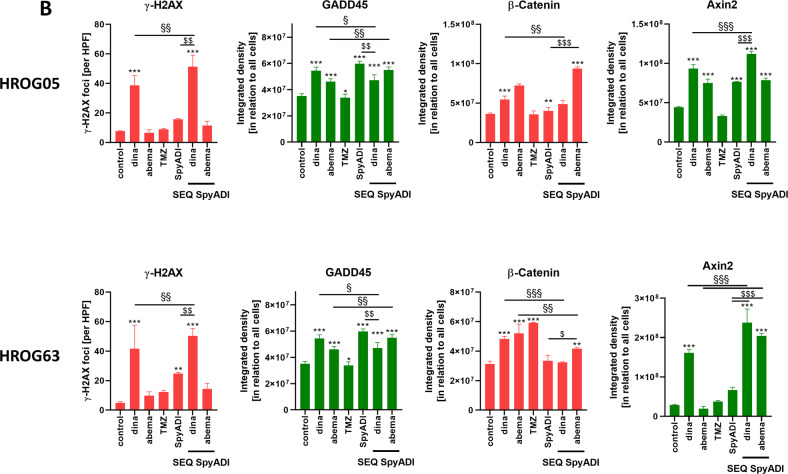
DNA-damage analysis using immunofluorescence and western blot. **A** Representative images of 2D-cultured GBM cells (HROG05, HROG63), treated with test substances for 2 × 72 h or left untreated. Cells were stained with the following monoclonal antibodies: anti-H2A.X Phospho (Ser139) [red], anti-GADD45 [green], anti-β-catenin [red], and anti-Axin22. For the latter, Axin2, a secondary DyLight 488-conjugated anti-rabbit antibody was applied [green]. Scale bar as indicated: 50 µm. Nuclei were counterstained with DAPI. Images were taken on a Zeiss Elyra 7 Confocal Laser Microscope. **B** Quantification of specific markers. *n* = 3 independent experiments; ***p < 0.001; §§*p* < 0.01; $$*p* < 0.01. One-way ANOVA.

Dinaciclib and SpyADI alone triggered DNA damage as indicated by significantly increased numbers of γ-H2AX foci and higher amounts of the Growth Arrest and DNA Damage-inducible 45 (GADD45) protein (Fig. 6A, B). In a direct comparison between HROG05 and HROG63, abundance of GADD45 was higher in in the latter. Dinaciclib and SpyADI, but not abemaciclib and TMZ stabilized GADD45 protein levels. SEQ-dinaciclib/SpyADI treatment had comparable, though slightly lower effects, mainly because of the high toxicity (Fig. 6A). Still, the amount of β-catenin, the key protein of the Wnt/β-catenin-signaling-pathway, was reduced in this combination, accompanied by significantly elevated levels of the β-catenin antagonist Axin2. Abemaciclib is a known inducer of β-catenin [19], a phenomenon also seen here upon monotherapy. SEQ-abemaciclib/SpyADI partially neutralized this effect—at least in HROG63, where β-catenin was significantly downregulated (vs. abema monotherapy, Fig. 6B). SpyADI alone had no impact on β-catenin but tended to induce Axin2. Interference with the Wnt/β-catenin-signaling-pathway is therefore anticipated.

### Combination treatment induces DNA-damage repair

Next, we checked for specific DNA repair proteins in HROG63 cells to underpin the above findings. SFig. 5 illustrates XRCC1 (repair of single-strand breaks), Ku70, Ku80, and Rad51 protein levels, most of them being responsible for DNA double-strand break repair. The monotherapies themselves altered the protein abundance of the respective targets. Combinations partially boosted effects, however, without reaching statistical significance. In detail, TMZ and SpyADI, but not CDKi monotherapy induced XRCC1. SEQ-CDKi/SpyADI enhanced XRCC1 protein levels, likely due to the high level of DNA damage induced by these combinations. Rad51 and Ku70 abundance differed significantly between individual therapies and was massively reduced by abemaciclib and/or with SpyADI. This effect was independent of SpyADI. By contrast, SEQ-dinaciclib/SpyADI triggered Rad51 and Ku80 to mediate homologous recombination and non-homologous-end-joining.

### Microarray analysis

**Fig. 7:**
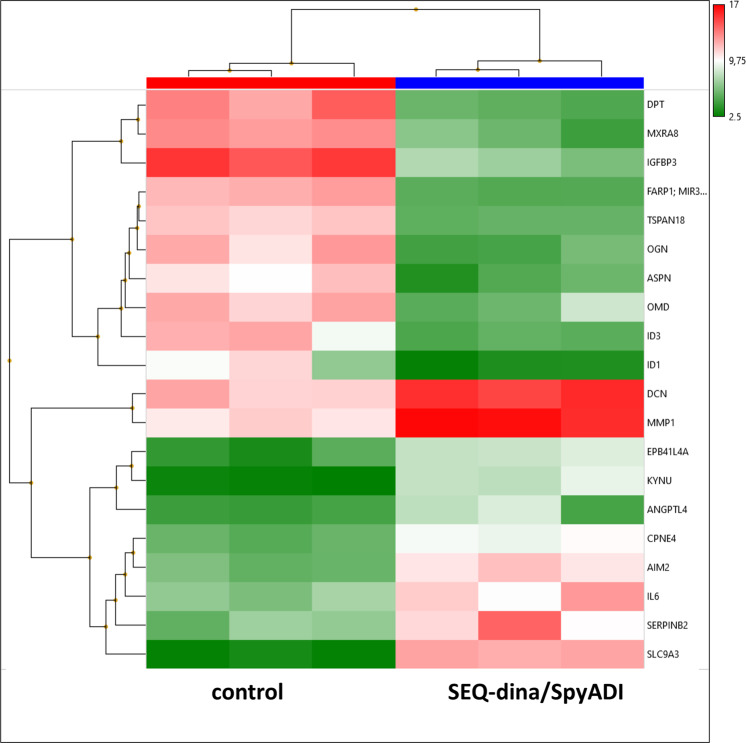
Heat map showing RNA expression level from 2D-cultured HROG63 cells. Therefore, cells were either treated with SEQ-dinaciclib/SpyADI or left untreated. RNA was isolated as described in material and methods and RNA expression level determined by applying Affymetrix Human Clariom S Array. Primary data analysis was performed with the Affymetrix TAC including the SST-RMA for normalization. Gene expression data were log-transformed. Limma was used here to calculate the *p*-value. A change was considered significant when the Limma eBayes *p*-value met the criterion *p* < 0.05 at fold-changes > |2 | , i.e., expression increments or declines larger than two. Data are collected from three independent experiments.

To explore the differential molecular properties, we finally performed a comparative microarray analysis of 2D-cultured HROG63 cells, either treated with SEQ-dinaciclib/SpyADI or left untreated (Fig. 7). Microarray analysis of 2D-cultured HROG63 cells treated with SEQ-dinaciclib/SpyADI (*vs*. control) identified 1727 altered genes with a fold-change of ≥2 and a *p*-value of < 0.05.

Most of the repressed genes (*DPT, MXRA8, TSPAN18, MGP, MFAP4, VCAM1*, and *EFEMP1*) are involved in cell adhesion, integrity of the extracellular matrix, and migration habits contributing to the cellular/cytoskeletal integrity. Three known oncogenes of the small leucine-rich repeat proteoglycan (SLRP) family (*ASPN, OGN, OMD*), were also downregulated. Additional repressed genes included those mediating transport (*APOD, SLC14A1*), transcription regulation (*ID3, ID1, KHDRBS3*), growth factor binding (*IGFBP3, IGFBP5*), differentiation (*APELA*), GTPase (*RERG*), and Rho-guanine nucleotide exchange factor (*FARP1*). By contrast, expression levels of known tumor suppressors, such as *EPB41L4A*, *SERPINB2, AIM2, NR4A3*, and *LUM*, but also those with relevance in tumor cell invasion, extracellular matrix remodeling, thus promoting oncogenic properties *(ICAM1, IL1RN, MMP1/3, DCN, HS3ST3B1, SEL1L3, STC1*, and *EEF1A2)*, were significantly increased (Fig. 7).

## Discussion

Abnormal regulation of the cell cycle and deregulation of cellular energetics are considered hallmarks of most tumors [20]. These processes are associated with the constitutive activation of various CDKs, as well as metabolic shifts depending on the presence of exogenous amino acids like arginine [21]. We previously demonstrated that the selective CDK4/6 inhibitor abemaciclib and the more global acting CDKi dinaciclib, as well as SpyADI, show cytotoxic activity as single agents against GBM due to different mechanisms [11, 16]. In the present study, we examined mechanistic insights and drug susceptibility of human GBM models treated with SpyADI and CDKis. To identify an optimal treatment regimen, different settings (order of application) and combination partners were applied. All CDKi/SpyADI combinations proved effective and decreased cell viability, colony formation, or invasiveness in 2D- and 3D models; the efficacy of SEQ-dinaciclib/SpyADI was often significantly higher than that of the other settings tested. An exception was seen for apoptosis/necrosis discrimination. Here, higher numbers of necrotic cells were seen under SEQ-abemaciclib/SpyADI treatment, while it did not reach statistical significance for the SEQ-dinaciclib/SpyADI regimen. There are several reasons explaining this discrepancy: (I) the cytotoxic potential of individual CDKIs is far more complex than solely inducing apoptosis/necrosis; (II) SEQ-dinaciclib/SpyADI is rapidly affecting cellular viability (vs. SEQ-abemaciclib/SpyADI) and this high toxicity leads to cell debris, which is easily washed away before measurement; and (III) residual cells show only modest signs of apoptosis/necrosis.

Notably, antitumoral effects of both CDKI/SpyADI combinations were boosted in clinically relevant 3D models. Indeed, viability, invasion, and growth of both glioma spheres and glioma stem-like cells was massively impaired. Given the fact that the latter is the main cause of recurrence and usually characterized as “drug-resistant”, this finding is of particular importance and highlights the clinical relevance of this novel treatment regimen based on SEQ-CDKI/SpyADI. Hence, we propose a combined approach in which CDKi are used in first-line followed by SpyADI in the 2nd round.

To get a more detailed look at drug-mediated cell death, we investigated the mode of action, focusing on 2D-cultures. Mitochondrial impairment characterized by elevated mitochondrial membrane potential, mito-ROS, and vacuole formation was already observed under CDKi monotherapy in our former study [16]. Here, we detected intensified mitochondrial membrane activity after CDKi-SpyADI combination regimens as well as after SpyADI monotherapy. Consistent with this, basal respiration and ATP-production-based respiration were reduced resulting in impaired energy metabolism. The complex effects of this combined treatment are accompanied by marked autophagy induction (abemaciclib-SpyADI > dinaciclib/SpyADI), corroborated by ultrastructural TEM analysis, showing mitochondrial structural damage, including treatment-dependent enlarged mitochondria. Furthermore, SpyADI and CDKi induced debris-filled vacuoles that may indicate impaired mitochondrial respiratory function, as previously described upon arginine starvation [11, 22–26]. Alternatively, such increased vacuolization may be related to other mechanisms of autophagy and cell death, as has been reported for mammalian cells, particularly after exposure to small molecules such as bacterial toxins [27]. Abemaciclib monotherapy induced small vacuoles and myelin figures, confirming lysosomal formation as well as induction of an atypical cell death closely resembling methuosis-like processes. Autophagy accompanied by regression has been described in myeloma cells and the latter in the lung cancer cell line A549 [28, 29]. In a parallel study on head and neck squamous cell carcinoma models, we detected a palbociclib-induced gain of mitochondrial activity and lysosomal formation [30]. Hence, the mechanisms induced by CDKi treatment are complex and far from being completely understood. Also, single amino acid deprivation by ADI is described to trigger autophagy and to impair mitochondrial respiratory function in cancer models [11, 22–26].

Dinaciclib treatment led to swollen ER and vacuoles likely originating from the ER. ER stress occurs due to an imbalance between ER protein-folding load and capacity under various physiological and pathological conditions. Here, ER stress was confirmed by slight increases in the chaperone calnexin and transcription factor ATF4 under dinaciclib and CDKi/SpyADI-combination therapy. Calnexin ensures proper folding and interacts with newly synthesized glycoproteins in the ER. ATF4 (CREB2) functions in the PERK/eIF2α ER stress-responsive pathway as an activating transcription factor/cAMP-response element-binding protein [31–33]. ER stress can also trigger UPR, to improve protein folding and assembly, thereby expanding the capacity of the ER to process proteins destined for the secretory pathway [34, 35]. Comparable mechanisms evoked by our CDKi/SpyADI treatment can be anticipated. Redistribution of cytochrome c was also seen in these regimens. Generally, arginine withdrawal activates ATF4 and asparagine synthetase (ASNS) via nutritional stress-mediated general control nonderepressible 2 activation and the ER stress pathway, which leads to increased consumption of aspartate. However, aspartate consumption is one reason for the cell death of arginine-auxotrophic cancer cells [26]. Also, flavopiridol, a pan-CDKi, and the multi-CDKi P1446A-05 were previously reported to (partially) induce ER stress and the UPR manifested by ASK1-dependent signaling in CLL [36, 37]. These results suggest that dinaciclib and the CDKi-SpyADI combination trigger ER stress in the form of swollen ER via ER chaperone and ATF4 activation in GBM cells.

Recent studies reveal critical roles for CDKs in regulating homologous recombination (HR) and non-homologous end-joining (NHEJ). However, CDK dysregulation is a hallmark of cancer cells and CDKs function as therapeutic targets [12, 38–43]. The phosphorylation of one of the variants of the nucleosome core histone H2A, namely, histone H2AX at Ser139 (γH2AX) occurs in response to the induction of DSBs, which is why γH2AX is considered a biomarker of DNA damage. Only recently, we reported DSBs and DDR induction after dinaciclib treatment—even without irradiation, likely contributing to genomic instability and histone alterations in GBM [16]. In this study, we observed changes in key components of the HR, NHEJ, DDR as well as repair of DNA single-strand breaks (Rad51, Ku70, Ku80, and XRCC1). Ku70 and Rad51 protein levels were massively reduced by abemaciclib. Dean *et al*. also demonstrated a Ku70 reduction in a human TNBC model under palbociclib treatment [44]. This implies that CDK4/6 inhibition may impair the ability of cancer cells to repair the induced DNA damage via NHEJ—as well as HR-mediated DNA repair. Treatment with dinaciclib and/or SpyADI revealed enhanced phosphorylation of histone γH2AX and a slight increase of Ku80 and Rad51. SpyADI and its combination with dinaciclib boosted repair incision activity and elevated HR, likely because of collapsing replication forks and increased DSBs. Then, we focused on GADD45, whose expression increases in stressful growth arrest conditions and/or treatment with DNA-damaging agents [45–47]. GADD45 is also a target of p53 and p21, so it is induced both with and without the help of p53. In most cancers, GADD45 functions are reduced and deregulation of regulators of GADD45 increases cell proliferation and tumorigenic potential [48]. Here, GADD45 was upregulated in HROG63 cells treated with dinaciclib and/or SpyADI. By promoting GADD45 transcription or transcript stabilization, arginine deprivation, and several chemotherapeutic agents like docetaxel, fucoxanthin induce GADD45 that contributes to survival impairment [49, 50]. We thus identified GADD45 activation complementary to increased γ-H2AX, Rad51, and Ku80 in GBM as another central switch to dinaciclib- and/or SpyADI-induced cell death.

The wingless or WNT pathway is implicated in development and stemness and has been associated with glioma stem-like cells and glioma pathobiology [51–53] in which growth behavior, WNT activity, and cytoplasmic/nuclear β-catenin correlate with AXIN2 expression and poor patient outcome [54]. A previous study on ovarian cancer cells reported decreased β-catenin level upon dinaciclib treatment [55]. Here, no such alteration was seen, with levels remaining unchanged under dinaciclib and/or SpyADI treatment, while AXIN2 increased. In contrast, abemaciclib+/– SpyADI induced β-catenin, likely because of abemaciclib-mediated WNT activation via inhibition of GSK3β [19]. Further studies are needed to determine if WNT activation also occurs in patients. Arginine epigenetically modulates the WNT/β-catenin pathway [56] and ASS1 promotes gastric cancer cell invasion through β-catenin stabilization [57]. We identified GADD45 induction upon therapy and thus hypothesize that SpyADI-induced arginine deprivation in combination with CDK-blockage increases AXIN2, which in turn degrades β-catenin to finally impair cell proliferation and induce senescence via p21 and DSBs. In support of this, genes involved in cellular/cytoskeletal integrity were significantly downregulated under therapy, while tumor suppressors were induced. Further underlying mechanisms include rather low participation of UPR and senescence. Abemaciclib-SpyADI-treatment suppressed the DSB repair system via NHEJ and HR, dinaciclib-SpyADI-treatment enhanced γ-H2AX accumulation and induced Rad51/Ku80. This latter combination activated the stress sensor GADD45 and β-catenin antagonist AXIN2. Thus, it is tempting to speculate that the common interaction between the dropped intracellular arginine level and CDK blockage affects p53, which in turn triggers DNA damage-related proteins and impairs mitochondrial activity to induce cell death. Our observations strengthen and redefine the dynamic and synergistic role of SEQ-CDKi-SpyADI in the treatment of Arg-auxotrophic GBM cases.

To the best of our knowledge, this is the first report on a novel combined therapy approach based on targeting two essential mechanisms in cancer: cell cycle and metabolism. By applying CDKis (abemaciclib/dinaciclib) and SpyADI sequentially, we could show that cell proliferation, invasiveness, gene expression, and viability of Arg-auxotrophic GBM cells is impaired, which is even pronounced in 3D-cultured stem-like cells. Combined CDKi/SpyADI induces massive mitochondrial impairment and vacuole formation, initially triggered by CDKi and boosted upon arginine deprivation. The observation of an amplified antitumoral response in clinically relevant 3D models emphasizes the potential of this approach. A very recent publication described quiescent GBM cancer stem cells that become activated upon chemotherapy [58]. Subsequent studies on organoid cultures will show whether our combined CDKi/SpyADI has the capacity to catch these “quiescent” GBM stem cells by counteracting their potential to evade anti-proliferative therapies.

## Materials and methods

### Patient-derived GBM tumor cell lines, normal control cells, and culture conditions

Patient-derived GBM cell lines (*n* = 3; HROG02, HROG05, HROG63) were established in our lab and basically characterized, including molecular analysis, MGMT promoter methylation status and ASS or ASL status [17, 18, 59]. 2D cell culture was done in full medium and incubated at 37 °C in a humidified atmosphere of 5% CO_2_: Dulbecco’s modified eagle medium: nutrient mixture F-12 supplemented with 10% FCS, l-glutamine (2 mmol/l) and antibiotics (100 U/ml penicillin/100 μg/ml streptomycin) (all from Pan Biotech, Aidenbach, Germany). 3D glioma spheres and glioma stem-like cells were cultured in ultra-low attachment (ULA) plates (Greiner Bio-One, Kremsmünster, Austria) in defined medium as described in [16]. Non-malignant cells included murine fibroblasts (L929), normal human dermal fibroblasts (NHDF), and human mesenchymal stem cells (h-MSC). L929 cells were cultured in DMEM full medium as described above, NHDF cells were cultured in Fibroblast Growth Medium (Promocell, Heidelberg, Germany), and h-MSC were cultured in a mixture of DMEM/IMDM + supplements (45% Iscove’s Modified Dulbecco’s Medium, 45% Gibco® F-12 Nutrient Mixture, 10% newborn calf serum (all from Thermo Fisher Scientific GmbH, Germany) supplemented with 10 μg basic fibroblast growth factor (Millipore Merck KGaA, Germany)). Additionally, HROG63 cells were transduced to yield stable expression of the fluorescent near-infrared protein iRFP680 (NIR). Correlation between cell death and loss of NIR-fluorescence was confirmed with DAPI staining and flow cytometry (*unpublished own data*).

### Cytostatic drugs and *S. pyogenes* arginine deiminase (SpyADI)

Drugs used in this study included the CDKis (Selleckchem, Munich, Germany) dinaciclib (10 or 100 nM) and abemaciclib (10 µM) and the standard therapeutic TMZ (10 µM, MSD, Haar, Germany). Arginine deiminase from *S. pyogenes* serotype M49 strain 591 (SpyADI, 35 mU/ml) was heterologously expressed in *E. coli DHα*, purified and activity was measured as reported previously [18, 60].

### Viability assay

Cell viability was assessed after 2 x 72 h by Calcein Acetoxymethyl (Calcein AM) (Biomol GmbH, Hamburg, Germany) fluorometric assay in 2D-culture (GBM, non-malignant control cells) as described in [16, 61]. 3D-cultures (spheroids) were analyzed luminometric using the CellTiter-Glo® 3D cell viability assay (Promega, Walldorf, Germany) according to the manufacturer’s instruction. CellTiter-Glo® 3D luminescence signal was read with a microplate reader (Infinite® M200, Tecan Group, Switzerland).

### MitoTracker® Red CMXRos, LysoTracker ™ Green DND-26, acridine orange, and X-gal staining

MitoTracker Red CMXRos. LysoTracker Green (Cell Signaling Technology; Thermo Fisher Scientific) and senescence-associated β-galactosidase (SA-β-gal, Cell Signaling Technology, Cambridge, UK) were stained as stated in [11, 16]. Images were taken on a fluorescence microscope (Leica DMI 4000B, Leica, Heidelberg, Germany). Besides, cells were stained with acridine orange (4 mg/ml; Applichem, Darmstadt, Germany) and Calcein AM (2 μM; Biomol, Hamburg, Germany) for 10 min at room temperature according to ref. [11]. Slides were analyzed on a Zeiss Elyra 7 Confocal Laser Microscope. Integrated density was quantified using ImageJ software.

### Cellular metabolic analysis

The OCR and ECAR were measured using the Seahorse Bioscience XFe24 Extracellular Flux Analyzer (Agilent Technologies, Inc., Waldbronn, Germany) according to the manufacturer’s instructions. In brief, the cells were seeded in Seahorse 24XFe plates with a density of 10,000 cells per well and treated as described above. One hour before Seahorse XFe analysis, the medium was changed to unbuffered XF base medium, pH 7.4 containing 10 mM glucose, 1 mM pyruvate, and 2 mM glutamine. The mitochondrial function was assessed by sequential injections of Seahorse XF Cell Mito Stress Test Kit compounds (Agilent Technologies, Inc.): via port A oligomycin to final concentration 1.5 µM, port B 1.0 µM FCCP, and port C 0.5 µM rotenone and antimycin A, respectively. Calculation of basal respiration, ATP-production-based respiration, proton leak and coupling efficiency was performed using the Seahorse XF Cell Mito Stress Test Report Generator (Agilent Technologies, Inc.). To normalize the data, the amount of protein per well was determined using the BCA Protein Assay Kit (Thermo Fisher Scientific Inc.). For this purpose, lysates were generated with lysis buffer (50 mM HEPES, 150 mM NaCl, 1 mM EDTA, 1% [v/v] Triton® X-100, 10% [v/v] glycerol, 5.2 µl/ml aprotinin, 2 µl/ml leupeptin, 10 µl/ml orthovanadate, 10 µl/ml PMSF).

### Immunofluorescence and western blot

GBM cells were treated for 2 x 72 h and further treated as described [16]. Additionally, CDKN2A/p16INK4a antibody (JC8) (1:50, Santa Cruz), Alexa Fluor® 488 p21 Waf1/Cip1 (1:300, Cell Signaling), Alexa Flour® 594 anti-p53 antibody (1:50, Biolegend), Alexa Fluor® 594 anti-H2A.X Phospho (Ser139) antibody (1:100, Biolegend), FITC anti-GADD45 antibody (1:100, Biorbyt), PE anti-β-Catenin antibody (1:75, Biolegend), anti-AXIN2 antibody (1:50; Thermo Scientific), Alexa 647 anti-ATF-4 antibody (B-3, 1:50, Santa Cruz), Alexa 594 anti-calnexin antibody (AF18, 1:50, Santa Cruz), and Alexa 488 anti-cytochrome c (1:50, Biolegend). For AXIN2, a secondary DyLight 488-conjugated anti-rabbit antibody was applied (1:100; Biolegend). Nuclei were counterstained with DAPI. Cells were analyzed on a Zeiss Elyra 7 Confocal Laser Microscope. Protein extraction and western blotting was done as described [11]—a list of antibodies is given in STable 1. All data sets were normalized to GAPDH, which was used as housekeeping control (please see SFig. 6 for original western blots).

### Colony formation assay

Cells were seeded in a 6-well plate at a density of 1.0 × 10^3^ per well and allowed to attach for 24 h. Adherent cells were treated as indicated for 2 × 72 h, with a 14-day follow-up. Cells were fixed with methanol, stained with 0.2% crystal violet and colonies photographed. Quantification of cell colonies was done using ImageJ software. Single colonies of treated cells were counted, however, the control was unable to count (>100 colonies/well) and is thus not presented in the graph.

### Tumor-spheroid invasion assay

After sphere formation (*n* = 1 sphere/well, *n* = 3 technical replicates x *n* = 3 biological replicates), 96-ULA well-plates were supplemented with reagents at a 2-fold final concentration (+epidermal growth factor to stimulate invasion) containing ice-cold matrigel (Corning, New York, USA) for 72 h. Then, spheres were treated again for 2 × 72 h and monitored for 15 days. Images were taken at a 5-day interval (Leica DMI 4000B). A detailed treatment schedule is presented in Fig. 3C.

### Transmission electron microscopy (TEM)

The cells were centrifuged at 300 × *g* for 5 min, supernatants were discarded, fixed with fixation buffer (1% paraformaldehyde, 2.5% glutaraldehyde, 0.1 M sodium phosphate buffer, pH 7.3), and stored at 4 °C. Further processing of specimens was performed as described previously [62]. Briefly, cell pellets were collected in low melt agarose, postfixed with osmium tetroxide, dehydrated and embedded in Epon resin. Thin sections of ~70 nm were contrasted with lead citrate and uranyl acetate and were examined with a Zeiss EM902 electron microscope operated at 80 kV Carl Zeiss Microscopy, Jena, Germany).

### Microarray analysis

RNA of treated and control HROG63 cells (5 × 10^5^ cells/treatment) was extracted, total RNA was quantified, and expression profiling was done using Applied Biosystem^TM^ Clariom^TM^ S arrays (formerly Affymetrix, Thermo Fisher Scientific) as described [16, 63].

### Image processing

Images quantification was done by using the FIJI-ImageJ software as follows: Images were split into respective channels via ZEN software (Zeiss, Oberkochen, Germany). Staining intensity was then determined by integrated density profiles of the same size.

### Statistics

All values are given as mean ± SD. Differences between controls and treated cells were determined by using one-way ANOVA after proving the assumption of normality (Tukey’s Multiple Comparison post hoc). In addition to testing the significance against the respective controls, differences between mono- and combination treatments were determined. Significant differences are marked as follows: *vs. control; §vs. CDKi monotherapy; $vs. SpyADI monotherapy; #vs. SIM-combination. Statistical evaluation was performed using GraphPad PRISM software, version 8.02 (GraphPad Software, San Diego, CA, USA). The criterion for significance was taken to be *p* < 0.05.

## Supplementary information

supplementary information

sFig. 1

sFig. 2

sFig. 3

sFig. 4

sFig. 5

STable 1

Reproducibility checklist

## Data Availability

All data generated or analyzed during this study are included in this published article (and its supplementary information files).
