# Supplementary material for: Implementation of a combined CDK inhibition and arginine-deprivation approach to target arginine-auxotrophic glioblastoma multiforme cells
Source: Cell Death Dis. 2022 Jun 18;13(6):555. doi: 10.1038/s41419-022-05006-1 (PMC9206658; doi:10.1038/s41419-022-05006-1)
Supplement: Supplementary file 1 — supplementary information [file 41419_2022_5006_MOESM1_ESM.docx]

**Supplementary information**

**Supplementary Figure 1:** GBM cells were treated with the respective substances for 2x72 h in a simultaneous or sequential treatment regimen (SIM; SEQ). Images are representative for the different treatment regimens in HROG05 and HROG63 cells cultured in 2D and 3D (scale bar: 50 μm).

**Supplementary Figure 2:** Combined analysis of spheroid viability and invasive capacity into a matrigel-matrix. Stably transduced HROG63-NIR680 glioma spheres were treated with indicated substances or left untreated. Glioma spheres were monitored for a total of 15 days. Images were taken at a 5-day interval using a Zeiss microscope Axio Observer 7 (Zeiss, Oberkochen, Germany) (scale bar A, B: 500 μm). (A, B) Representative images of HROG63-NIR680 glioma spheres. (A) Red fluorescence indicates cellular viability. (B) Merged images resulting from phase contrast and red fluorescence to study invasion and viability simultaneously. (C) Viability was quantified using via the integrated density analysis with ImageJ software.

Invasive capacity (sphere area [µm^2^]) was examined with the oval selection function of FIJI-ImageJ according to ^64^. Finally, a ratio of invasion and viability was calculated to quantify the number of viable invading cells per spheroid. * p<0.05, *** p< 0.001, **** p<0.0001; $ p<0.05; $$$$ p<0.0001. Two-way ANOVA.

**Supplementary Figure 3: Quantification of molecular machinery of autophagy.** To further address molecular changes resulting in autophagosome formation GBM cells were treated as indicated, proteins collected and quantification of autophagy antibody sampler kit in relation to GAPDH was conducted. n = 3 independent experiments. One-way ANOVA was used.

**Supplementary Figure 4: Senescence analysis.** (A) Cells were fixed and stained with β-galactosidase solution overnight at 37 °C without CO_2_. Blue: ß-galactosidase-activity, indicative for senescence. Images taken on a Leica DMI 4000B microscope (scale bar: 50 μm). (B) HROG05 cells demonstrated treatment-associated increased levels of p53 and p21; p16 remained mainly unaffected. GBM cells were treated as indicated, fixed, permeabilized, and stained with p53 antibody [red], p21 antibody [green] and p16 [orange]. Nuclei were counterstained with DAPI (scale bar: 50 μm). Images were taken on a Zeiss Elyra 7 Confocal Laser Microscope. Data are collected from three independent experiments.

**Supplementary Figure 5: DNA damage analysis using western blot**. GBM cells were treated with the respective substances for 2x72 h in a sequential treatment regimen. Quantitative analysis of protein abundance (XRCC1, RAD51, Ku70/80) was examined in relation to GAPDH. n=3 independent experiments; * p <0.05; ** p < 0.01; $$ p<0.01.

**Supplementary Figure 6: Original western blots**. GBM cells were treated with the respective substances for 2x72 h in a sequential treatment regimen. Proteins were isolated as stated in material and methods and western blots were performed as described in ^11^ – a list of antibodies is given in STable 1.
