# Supplementary figures and images for: Implementation of a combined CDK inhibition and arginine-deprivation approach to target arginine-auxotrophic glioblastoma multiforme cells
Source: Cell Death Dis. 2022 Jun 18;13(6):555. doi: 10.1038/s41419-022-05006-1 (PMC9206658; doi:10.1038/s41419-022-05006-1)

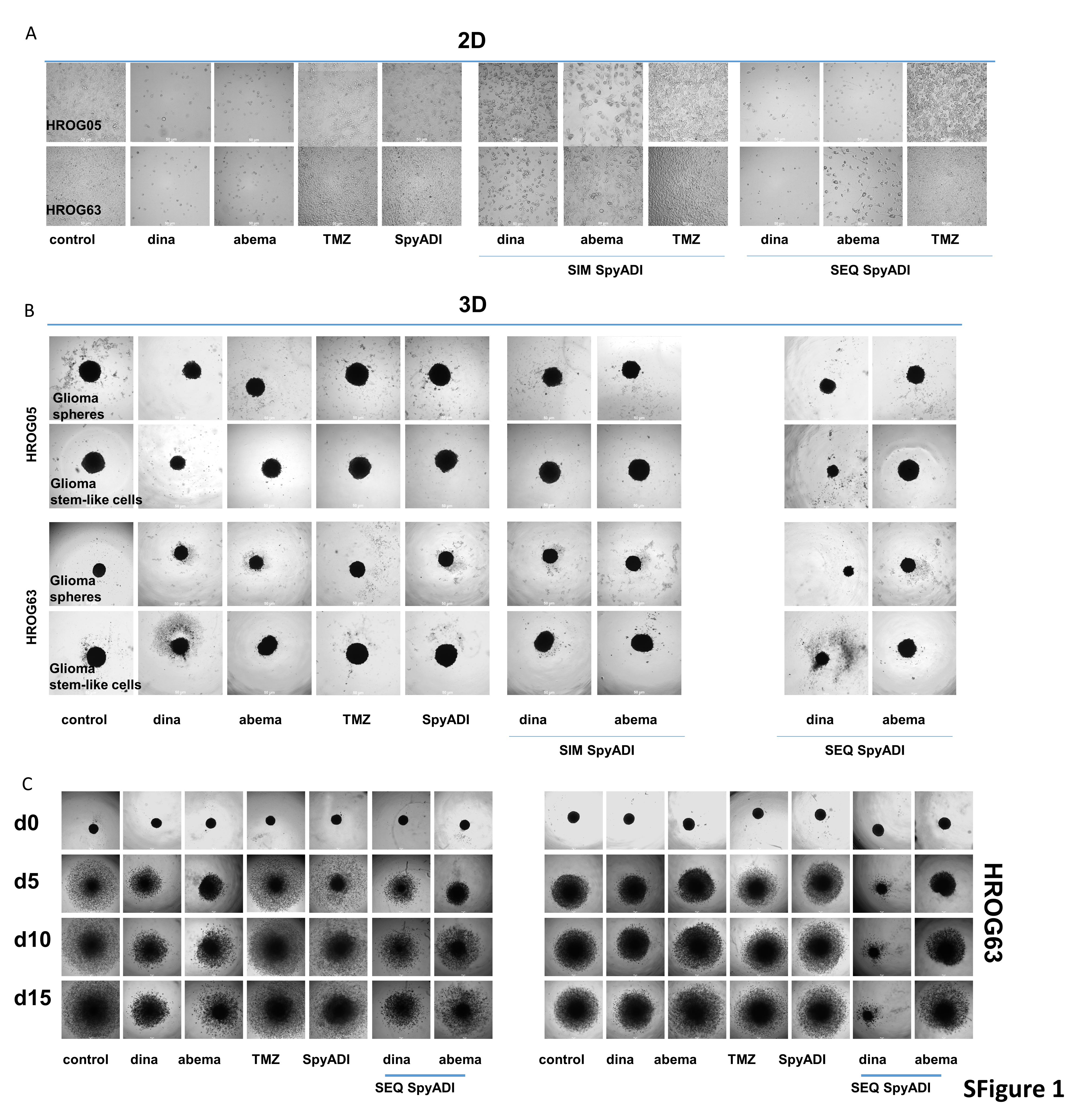

Supplement: Supplementary file 2 — sFig. 1 [file 41419_2022_5006_MOESM2_ESM.tif]

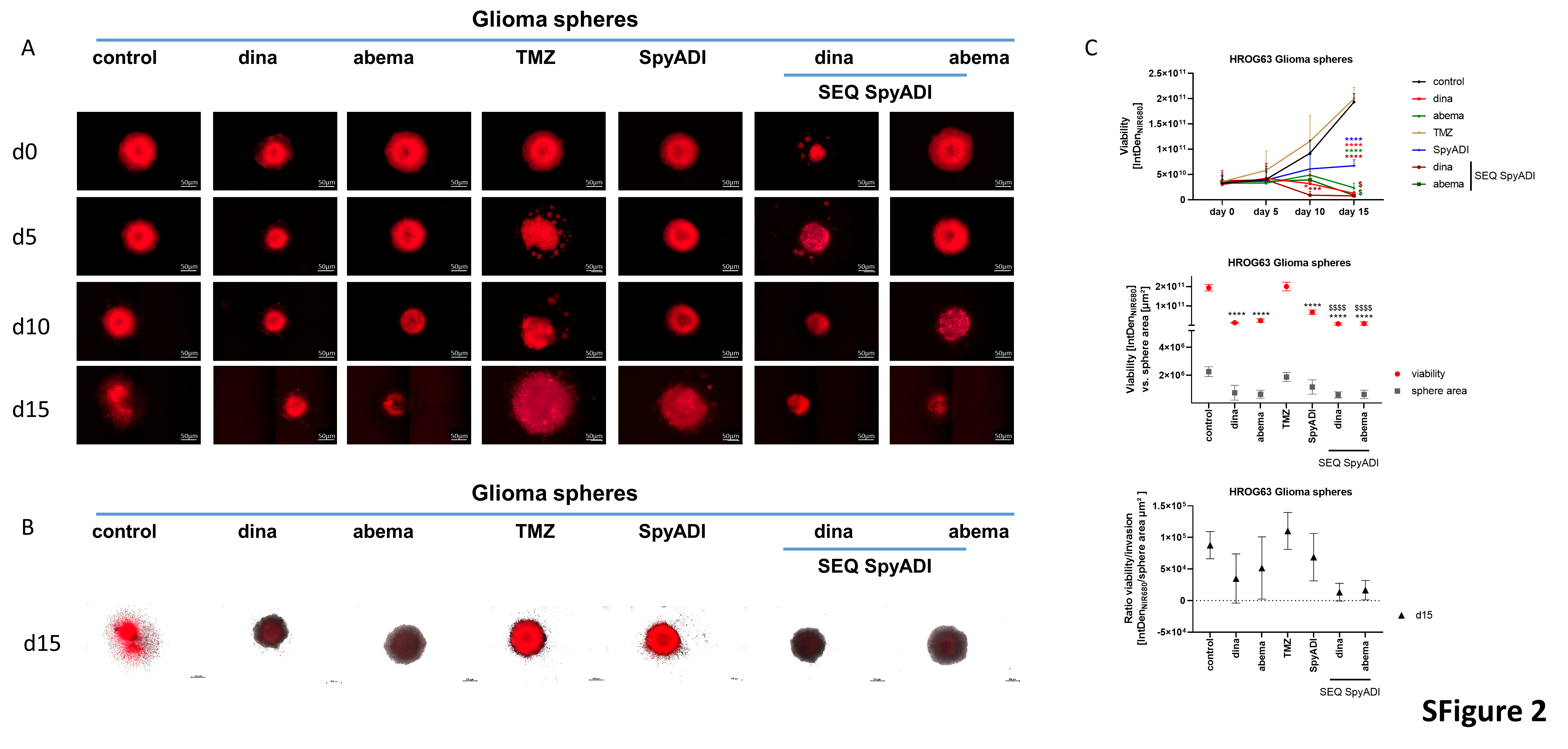

Supplement: Supplementary file 3 — sFig. 2 [file 41419_2022_5006_MOESM3_ESM.tif]

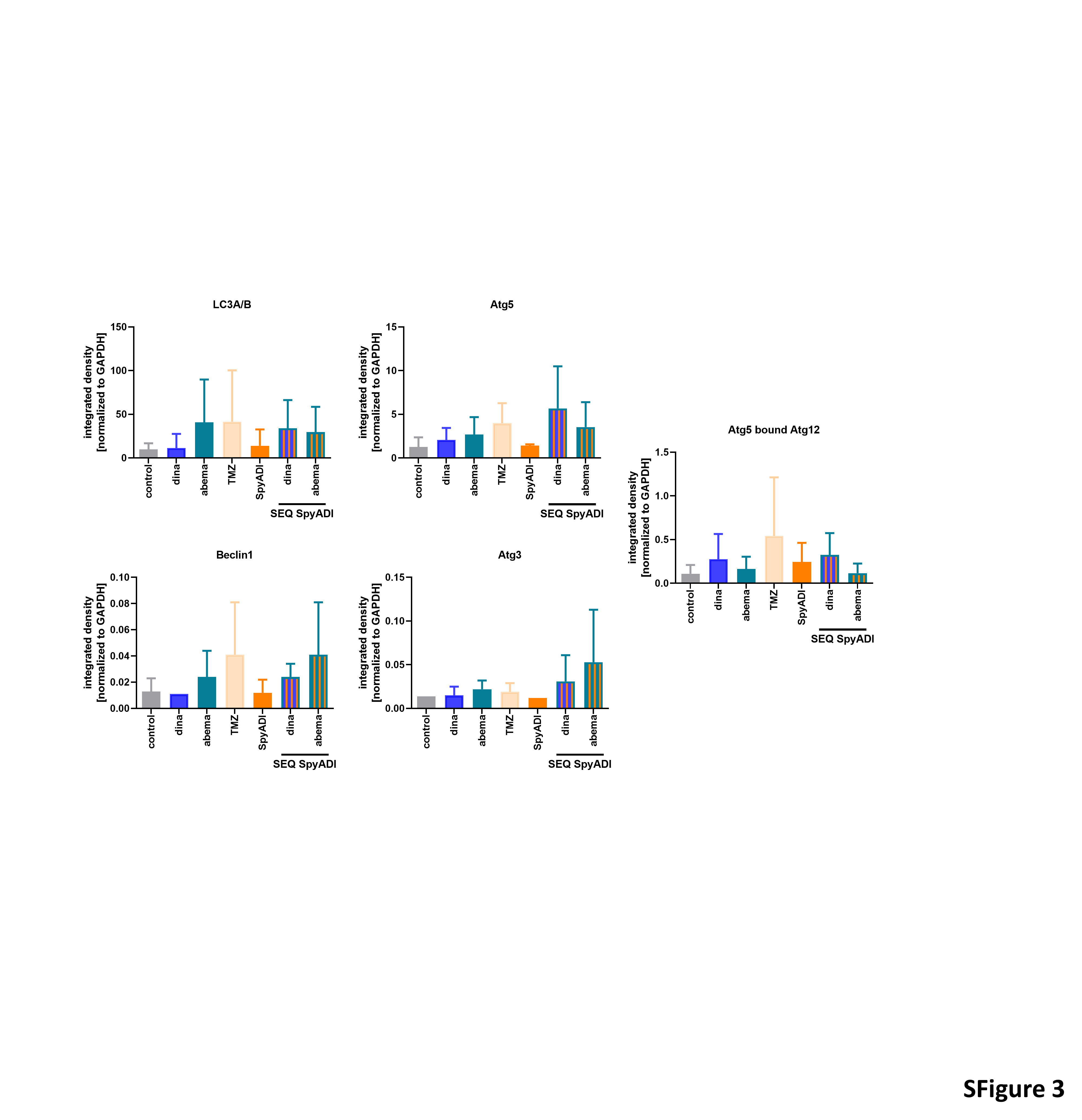

Supplement: Supplementary file 4 — sFig. 3 [file 41419_2022_5006_MOESM4_ESM.tif]

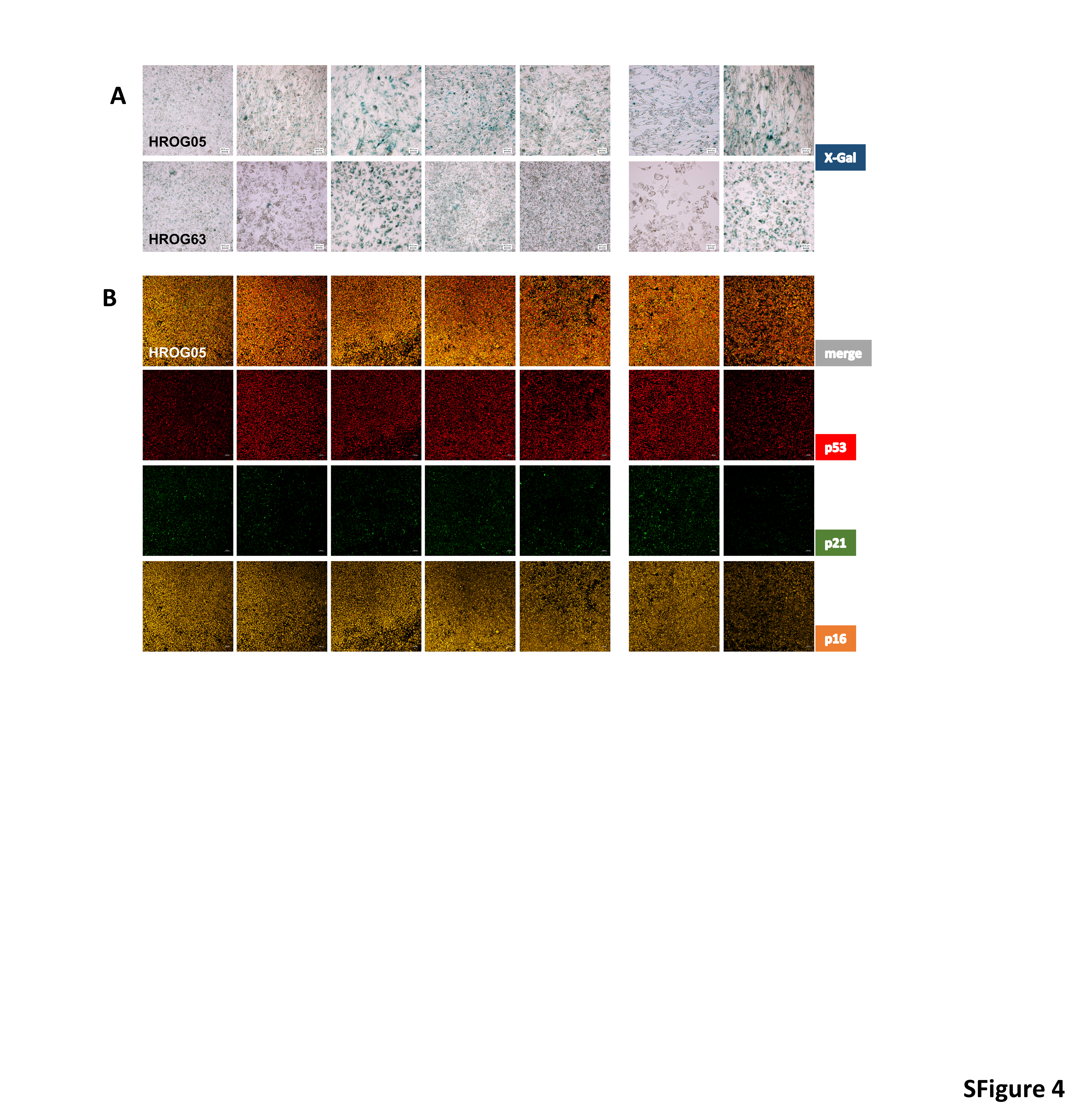

Supplement: Supplementary file 5 — sFig. 4 [file 41419_2022_5006_MOESM5_ESM.tif]

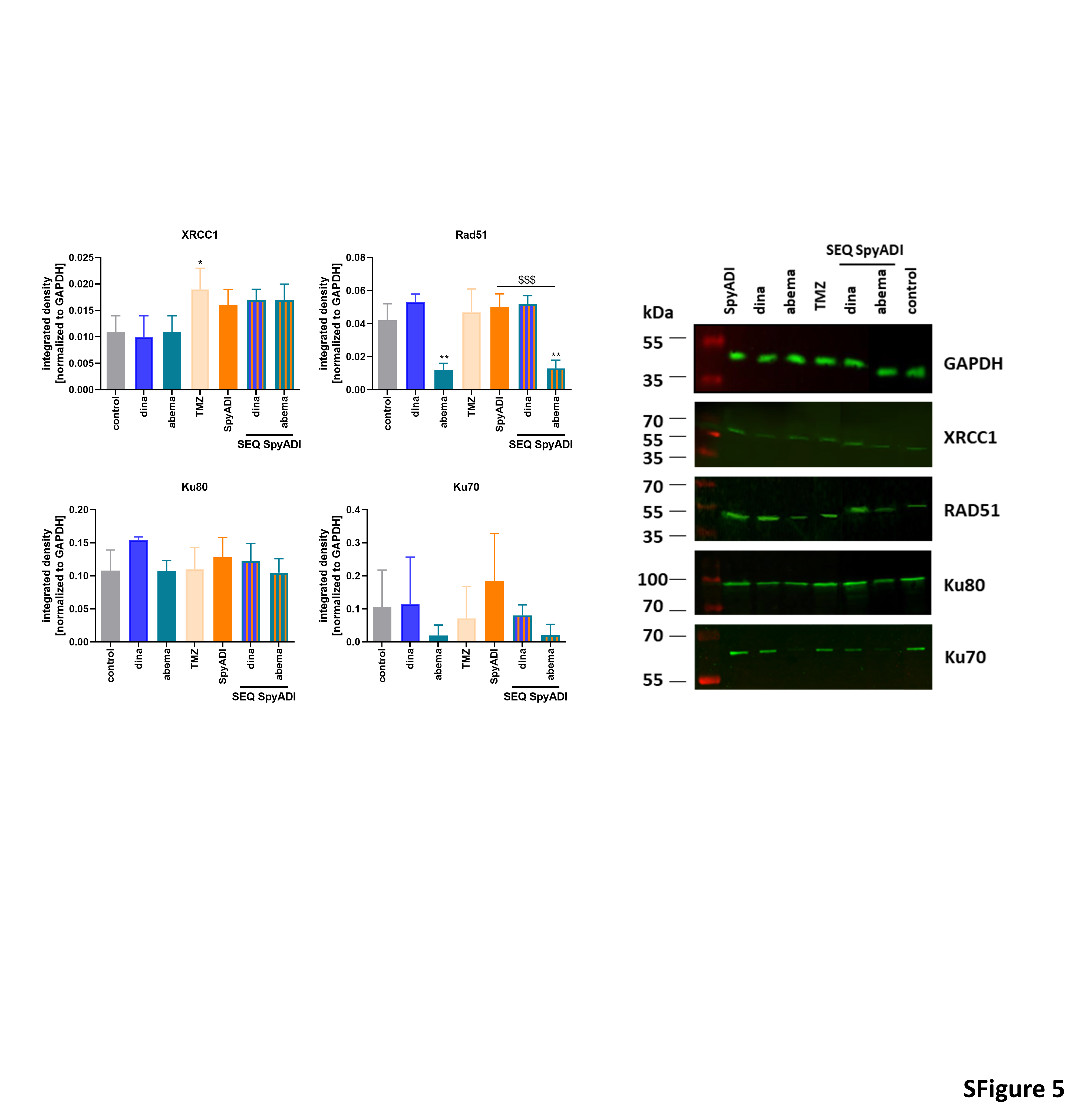

Supplement: Supplementary file 6 — sFig. 5 [file 41419_2022_5006_MOESM6_ESM.tif]
