# Supplementary material for: Implementation of a combined CDK inhibition and arginine-deprivation approach to target arginine-auxotrophic glioblastoma multiforme cells
Source: Cell Death Dis. 2022 Jun 18;13(6):555. doi: 10.1038/s41419-022-05006-1 (PMC9206658; doi:10.1038/s41419-022-05006-1)
Supplement: Supplementary file 7 — STable 1 [file 41419_2022_5006_MOESM7_ESM.docx]

**Supplementary Table 1. Antibodies used in this study.**

| Molecule | Cat number | Company | Species | Dilution |
| --- | --- | --- | --- | --- |
| p16 | sc-56330 AF546 | Santa Cruz | Ms | x 50 |
| p21 | 5487S | CST | Rb | x 300 |
| p53 | 645706 | biolegend | Ms | x 50 |
| γ-H2A.X (Ser139) | 613410 | biolegend | Ms | x 100 |
| GADD45 | orb15637 | biorbyt | Rb | x 100 |
| β-Catenin | 844606 | biolegend | Ms | x 75 |
| AXIN2 | MA5-32646 | TS | Rb | x 50 |
| ATF-4 | sc-390063 AF647 | Santa Cruz | Ms | x 50 |
| Calnexin | sc-23954 AF594 | Santa Cruz | Ms | x 50 |
| Cytochrome c | 612308 | biolegend | Ms | x 50 |
| DyLight 488 | 406404 | biolegend | Dk | x 100 |
| GAPDH | 39-8600 | Invitrogen |  | x20.000 |
| Ku70 | Sc-5309 | Santa Cruz | Ms | x200 |
| Ku80 | MA5-12933 | TS | Ms | x200 |
| XRCC1 | MA5-13412 | TS | Ms | x200 |
| Rad51 | MA5-14419 | TS | Ms | x200 |
| Autophagy Sampler Kit | 4445T | CST | as indicated in manual | x200 |
| IRDye secondary antibody700/800 | C50113-06 | Li-cor | Gt | x10.000 |
| CST, Cell Signaling Technology; Thermo Scientific, TS; Rb, rabbit; Ms, mouse; Rt, rat; Dk, Donkey; Gt, Goat; | | | | |
